# Effect of Chlorine and Temperature on Larvicidal Activity of Cuban *Bacillus thuringiensis* Isolates

**Published:** 2019-03-30

**Authors:** Aileen González-Rizo, Camilo E Castañet, Ariamys Companioni, Zulema Menéndez, Hilda Hernández, M Magdalena-Rodríguez, Rene Gato

**Affiliations:** 1 Departamento Control de Vectores, Centro de Investigación Diagnóstico y Referencia, Instituto de Medicina Tropical “Pedro Kourí”, La Habana, Cuba; 2 Facultad de Biología, Universidad de La Habana, La Habana, Cuba; 3 Departamento de Parasitología, Centro de Investigación Diagnóstico y Referencia, Instituto de Medicina Tropical “Pedro Kourí”, La Habana, Cuba

**Keywords:** Mosquitoes, Biological control, *Bacillus thuringiensis*, Bioassays, Chlorine

## Abstract

**Background:**

The efficacy of biolarvicides may be influenced by species of mosquito, larval age and density, temperature, water quality, bacterial formulation, and others. The aim of this study was to evaluate the influence of temperature and chlorine on larvicidal activity of *Bacillus thuringiensis* Cuban isolates against *Aedes aegypti*.

**Methods:**

The influence of temperature (25, 30, 35 °C) and chlorine (2.25mg/L) on the larvicidal activity of eleven *B. thuringiensis* Cuban isolates (collected between 2007 and 2009) were tested under laboratory conditions following WHO protocols. Bioassay data were analyzed by Probit program. The effect of chlorine and temperature (25, 30, 35 and 40 °C) on the Cry and Cyt proteins of these isolates was determined by SDS-PAGE polyacrylamide gel electrophoresis.

**Results:**

The pathogenicity of the isolates U81, X48 was affected at 35 °C. However, A21, A51, L910, and R89 isolates increase their entomopathogen activity at 35 °C. No differences were observed in toxicity of M29, R84, R85 and R87 isolates at different temperatures. The Cry 4, Cry 10 and Cry 11 proteins were reduced in A21, X48, R85 isolates at 35 and 40 °C. The Cyt proteins were reduced at 35 and 40 °C in A21, X48, R85, and A51 isolates. In L910 and R84 isolates, the Cyt toxin was degraded only at 40 °C. In chlorinated water, the lethal concentrations 50 and 90 in A21, A51, M29, R84, U81, and X48 isolates were increase.

**Conclusion:**

A21, A51, L910, R85, and X48 isolates have a strong larvicidal activity for the treatment of *Ae. aegypti* breeding’s sites exposed to high temperature and chlorine.

## Introduction

Mosquitos from the genus *Aedes*, specifically *Ae. aegypti* (Linnaeus) and *Ae. albopictus* (Skuse) are responsible to the arbovirus transmission and are closely related to urbanized environments. For this reason, this genus has become the main vector of arboviruses in the world (1). Vector control is the best way to reduce the arbovirus’s incidence. The *Ae. aegypti* control should be directed to the elimination of immature and adults’ stages near to the urbanizing sites (2).

For many years, *Bacillus thuringiensis* formulations have been the most important biopesticide used for agriculture and health (3). They have the advantages of specificity, high efficiency and environmentally safe (4).

*Bacillus thuringiensis* is a gram-positive sporulated bacterium. This bacterium in limited nutrient conditions sporulates and produces parasporal crystals with natural insecticidal properties. These crystals showed specific toxicity against invertebrates of the orders: Lepidoptera, Diptera, Coleoptera, some nematodes, mites, and protozoa (5).

Thousands of *B. thuringiensis* strains have shown considerable variability in their insecticidal toxicity. However, only highly toxic strains are used for biopesticide production (3).

Different factors like temperature and water quality affect the larvicidal effect of *B. thuringiensis* suspension (6, 7). This lack of residual activity is due to the low stability of its Cry and Cyt toxins and the poor recycling of spores under field conditions (6, 7). Therefore the present study assessed the influence of temperature and water chlorination on the larvicidal activity of eleven *B. thuringiensis* Cuban native isolates (8, 9) in order to select the better isolates to control *Ae. aegypti* larvae in breeding sites.

## Materials and Methods

### Reference Strains

*Bacillus thuringiensis* serotype H-14: IPS 82 from the International Entomopathogenic *Bacillus* Centre, Institute Pasteur; Paris, France.

Mosquitoes: *Ae. aegypti* (Rockefeller strain). Mosquitoes were reared in 18× 18× 18 inch collapsible cages (Bio Quip, USA) maintained at 26 °C±0.5 °C in 80–85% relative humidity (RH) with a photoperiod of 12: 12h (light/dark). A continuous supply of sucrose solution was provided. Female mosquitoes were given access to an anesthetized mouse and allowed to blood feed for 30min weekly. The larvae were fed finely powdered fish food.

### *Bacillus thuringiensis* isolates

A21, A51, L95, L910, M29, R84, R85, R87, R89, U81, X48 Cuban native isolates from soil samples of Cuba collected between 2007 and 2009 (8, 9). These isolates belongs to the entomopathogenic bacteria collection from the biological control laboratory of the Tropical Medicine Institute, “Pedro Kourí”, IPK, Cuba.

### Bacterial formulations

The bacterial isolates and reference strain were inoculated in a fermentation medium consisted of sucrose (2g/L), bacteriological peptone (2g/L), yeast extract (1g/L), and inorganic salts (12.5mmol/L MgSO4, 0.05mmol/L MnSO4, 1.2mmol/L FeSO4, 1.2mM ZnSO4, 25mmol/L CaCl2), incubated at 30 °C 48–72h at 150rpm (Retomed, Cuba). The bacterial sporulation was monitored through microscope. When more than 90% of cells lysed, the sporulated broth culture was transferred to 4 °C considered the final product (FP). Concentrations were expressed in mg/L (dry weight).

### Larvicidal efficacy

Quantitative bioassays were conducted following WHO protocol (10). Twenty-five larvae (III–IV instar) were introduced into 120mL cups with 100mL of dechlorinating water. Four replicates per dose were included and the experiments were repeated at least three times. Five concentrations of FP that cause mortalities between 10% and 90% were accepted for validating the bioassay. Mortality data were recorded after 24h of exposure and were used to calculate the lethal concentrations for 50% and 90% of exposed individuals (LC_50_ and LC_90_, respectively) through log-probit analysis (11). Abbott formula was used and the survival percentages were corrected if necessary (12). These values were compared to those obtained for the reference strain in order to estimate the efficacy of each isolate. The means of larval mortality caused by each isolate against *Ae. aegypti* were calculated. A value of P< 0.05 was considered statistically significant. Once the lethal doses were calculated, the CL_95_/CL_50_ ratio was performed to determine how many times it is necessary to increase the LC_50_ in order to obtain higher mortality. A lower ratio is indicative of better formulation efficiency (13).

Effect of temperature on larvicida activity: taking into account the temperatures average in Cuba (14). Larvicidal efficacy bioassays were performed at temperatures of 25, 30 and 35 °C.

The effect of chlorine on larvicide efficacy: the water for larvicidal efficacy bioassays was treated with sodium dichloroisocyanurate resulting in chlorine concentration of 2.25 mg/L, pH 6.8. The WHO guidelines on drinking water quality recommended not exceed the value of 5mg/L for free chlorine as sodium dichloroisocyanurate (15). The bioassays performed in dechlorinate water at 25 °C were used as control.

### The effect of abiotic factors on *Bacillus thuringiensis* Cry and Cyt proteins

Temperature treatment: In order to determine how the temperature affected the main virulence factors of *B. thuringiensis* native isolates. The FP of isolates was exposed to a range of temperatures 25, 30, 35 and 40 °C for 72h.

Chlorine treatment: The FP was treated with sodium dichloroisocyanurate resulting in chlorine concentration of 2.25mg/L, pH 6.0 and incubated at 25 °C for 24h.

Protein profiles: After each treatment (temperature and chlorine), the FPs were centrifuged (10,000 rpm for 20min) and the pellets were washed twice with 1mol/L NaCl and then with distilled water. The pellet was re-suspended in 100μL of distilled water and 100 of sample buffer (500mmol/L Tris-HCl pH 6.8, 10% SDS, 4% 2-mercaptoethanol, 8% glycerol, 0.1 % bromophenol blue), and boiled at water bath for 5min (16).

The protein profiles of the crystal components were determined by SDS-polyacrylamide gel electrophoresis (PAGE) (17) with 10% acrylamide separating gels. Tenμl of each sample was loaded onto a gel immediately before electrophoresis. FiveμL of a molecular weight marker (Broad Range Protein Molecular Weight Markers, Promega, USA) was added to each gel. The molecular weight of each protein was calculated with GelQuant software version 2.7.0 (Bio-Imaging Systems, Israel).

### Statistical analysis

Data from the evaluation of temperature were subjected to analysis of variance (ANOVA) and means were separated at the 5% significance level by using the Tukey HSD post-test. A log-transformation was used to calculate the slope values. The analysis of chlorine data was performed by *t*-Student test.

### Ethical approval

This study was carried out according to the principles expressed in the Declaration of Helsinki. The protocols were approved by the Institutional Research Ethical Committee at the Institute of Tropical Medicine “Pedro Kourí”.

## Results

### Influence of temperature on the toxicity of native isolates of *Bacillus thuringiensis* on *Ae. aegypti* larvae

The pathogenicity of the isolates U81 and IPS-82 were affected at 35 °C. However, the isolates A21, A51, L910, R84, R85, and R89 showed a significant improvement of their concentration lethal 90 (CL_90_) at 35 °C compared to the CL_90_ obtained at 25 °C (P< 0.05). However, only the R89 and X48 isolates improve their efficiency at high temperature. No differences were observed in toxicity of M29 and R87 isolates when bioassays were performed at different temperatures (Table 1).

**Table 1.** Entomopathogenic activity [lethal concentration (LC_50_ and LC_90_)] and efficiency of isolates of *B. thuringiensis* on *Aedes aegypti* at 25, 30 and 35 °C

**Isolates and strains**	**Variable**	**25 °C**	**30 °C**	**35 °C**
**A21**	LC_50_ (mg/L) (CL)	0.00374 (0.00329–0.00422)	0.00133 (0.00121–0.00145)	0.00070 (0.00062–0.00079)*
	LC_90_ (mg/L) (CL)	0.01278 (0.0106–0.01609)	0.00411 (0.00352–0.00508)*	0.00464 (0.0037–0.00606)*
	LC_95_ (mg/L) (CL)	0.01810 (0.01455–0.02400)	0.005704 (0.00487–0.00790)*	0.00790 (0.00603–0.01105)*
	Efficiency	4.84	4.29	11.2

**A51**	LC_50_ (mg/L) (CL)	0.00153 (0.00135–0.00173)	0.00070 (0.00061–0.00080)*	0.00002 (0.000001–0.00007)*
	LC_90_ (mg/L) (CL)	0.00455 (0.00374–0.00593)	0.001602 (0.00132–0.00211)*	0.00012 (0.00007–0.00030)*
	LC_95_ (mg/L) (CL)	0.00621 (0.00491–0.00855)	0.00226 (0.00202–0.03230)*	0.00035 (0.00023–0.00093)*
	Efficiency	4.05	3.74	13.78

**L95**	LC_50_ (mg/L) (CL)	0.09819 (0.08325–0.11700)	0.07864 (0.06237–0.10706)*	0.06140 (0.05490–0.069680)*
	LC_90_ (mg/L) (CL)	0.21750 (0.17275–0.30899)	0.38221 (0.23742–0.82760)	0.20240 (0.15830–0.28520)
	LC_95_ (mg/L) (CL)	0.2724 (0.23088–0.3247)	0.5983 (0.34174–1.5000)	0.9345 (0.83600–1.05940)
	Efficiency	2.77	7.6	15.22

**L910**	LC_50_ (mg/L) (CL)	0.01019 (0.00941–0.01093)	0.01010 (0.00808–0.01300)	0.00199 (0.00179–0.00219)*
	LC_90_ (mg/L) (CL)	0.01920 (0.01757–0.02185)	0.02119 (0.01577–0.03689)	0.00593 (0.00520–0.00695)*
	LC_95_ (mg/L) (CL)	0.02316 (0.0206–0.02706)	0.02614 (0.01864–0.0508)	0.00808 (0.00689–0.00989)*
	Efficiency	2.55	2.59	4

**M29**	LC_50_ (mg/L) (CL)	0.06569 (0.05833–0.07440)	0.05458 (0.04310–0.07440)	0.02447 (0.02111–0.03178)
	LC_90_ (mg/L) (CL)	0.13652 (0.11449–0.17510)	0.32005 (0.18700–0.83119)	0.06622 (0.05640–0.15280)
	LC_95_ (mg/L) (CL)	0.1678 (0.1368–0.2250)	0.5284 (0.2779–0.71686)	0.08746 (0.07544–0.11357)
	Efficiency	2.56	9.62	3.57

**R84**	LC_50_ (mg/L) (CL)	0.00954 (0.00862–0.10358)	0.00293 (0.00254–0.00337)	0.00284 (0.00244–0.00339)
	LC_90_ (mg/L) (CL)	0.01913 (0.01741–0.02162)	0.00717 (0.00588–0.00958)*	0.00803 (0.00610–0.01230)*
	LC_95_ (mg/L) (CL)	0.02573 (0.0235–0.0298)	0.00924 (0.00800–0.01219)	0.01080 (0.00779–0.01794)
	Efficiency	2.72	3.15	3.8

**R85**	LC_50_ (mg/L) (CL)	0.00793 (0.00697–0.00901)	0.00189 (0.00153–0.00220)	0.00107 (0.00091–0.00126)
	LC_90_ (mg/L) (CL)	0.01700 (0.01478–0.02176)	0.00496 (0.00410–0.00665)*	0.00507 (0.00359–0.00855)*
	LC_95_ (mg/L) (CL)	0.02170 (0.01792–0.02847)	0.00652 (0.00516–0.00959)*	0.00788 (0.00518–0.01498)*
	Efficiency	2.77	3.45	7.37

**R87**	LC_50_ (mg/L) (CL)	0.02897 (0.02616–0.03232)	0.00565 (0.00500–0.00643)	0.00253 (0.00199–0.00325)
	LC_90_ (mg/L) (CL)	0.07831 (0.06713–0.09530)	0.0105 (0.00886–0.01351)	0.01242 (0.00821–0.02462)
	LC_95_ (mg/L) (CL)	0.10400 (0.0866–0.13102)	0.0125 (0.0109–0.0151)	0.01950 (0.01182–0.0.04533)
	Efficiency	3.58	2.21	7.7

**R89**	LC_50_ (mg/L) (CL)	0.07146 (0.06444–0.07903)	0.01252 (0.00527–0.01637)	0.00529 (0.00302–0.00649)
	LC_90_ (mg/L) (CL)	0.17308 (0.14962–0.20820)	0.05094 (0.03350–0.27200)*	0.01191 (0.01020–0.04760)*
	LC_95_ (mg/L) (CL)	0.2223 (0.1873–0.27823)	0.07584 (0.043416–0.78746)*	0.01499 (0.00799–0.09271)*
	Efficiency	3.14	6.06	2.83

**U81**	LC_50_ (mg/L) (CL)	0.00461 (0.00424–0.00491)	0.00163 (0.00145–0.00181)*	0.00466 (0.00346–0.00683)
	LC_90_ (mg/L) (CL)	0.00905 (0.00810–0.01043)	0.00332 (0.00285–0.00409)*	0.04605 (0.02259–0.15460)*
	LC_95_ (mg/L) (CL)	0.01095 (0.0096v0.01304)	0.00406 (0.00339–0.00523)*	0.08895 (0.03814–0.3774)*
	Efficiency	2.37	2.48	19.08

**X48**	LC_50_ (mg/L) (CL)	0.00213 (0.00192–0.00236)	0.00079 (0.00060–0.00090)*	0.00222 (0.00200–0.00248)
	LC_90_ (mg/L) (CL)	0.00527 (0.00450–0.00643)	0.00094 (0.00082–0.00125)*	0.00585 (0.00486–0.00746)
	LC_95_ (mg/L) (CL)	0.00945 (0.00825–0.01127)	0.00108 (0.009212–0.01576)*	0.007677 (0.00619–0.01028)
	Efficiency	4.44	1.36	3.46

**IPS-82**	LC_50_ (mg/L) (CL)	0.00224 (0.00170–0.00273)	0.00067 (0.00061–0.00073)*	0.00567 (0.00490–0.00680)*
	LC_90_ (mg/L) (CL)	0.00567 (0.00457–0.00749)	0.00177 (0.00157–0.00207)*	0.02417 (0.01740–0.03865)*
	LC_95_ (mg/L) (CL)	0.00892 (0.00731–0.01174)	0.002128 (0.001805–0.00262)	0.0364 (0.0246–0.06371)*
	Efficiency	3.3	3.17	6.41

* P≤ 0.05

CL: 95% confidence limits

Efficiency: LC_95_/LC_50_

### Effect of temperature on protein profile

**Fig. 1.**
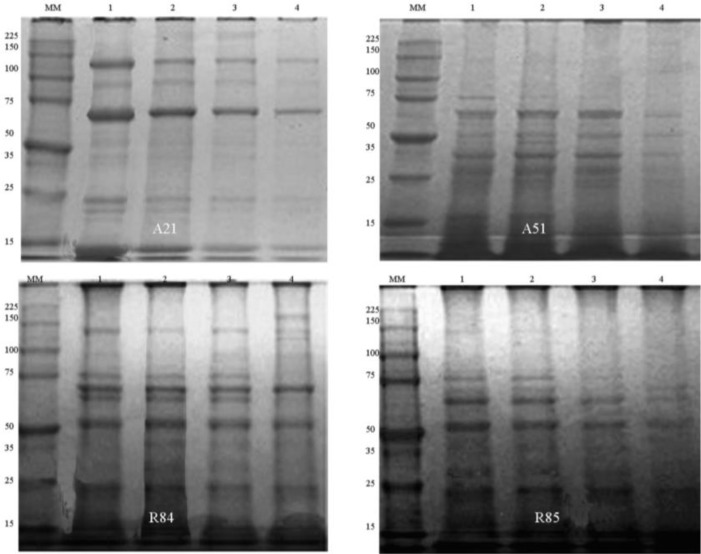
Analysis of Cry and Cyt protein of A51, A21, R85 and R84 isolates by SDS-PAGE (Coomassie brilliant blue-stained gel) Lane MM: Molecular weight Marker, Lanes-1: Proteins profiles of FP treated at 25 °C, Lanes-2: Proteins profiles of FP treated at 30 °C, Lanes-3: Proteins profiles of FP treated at 35 °C, Lanes-4: Proteins profiles of FP treated at 40 °C

The stability of these proteins at different temperatures (25, 30, 35 and 40 °C) was tested by SDS-PAGE (Fig. 1). All four bands corresponding with Cry and Cyt proteins were observed after treatment at 25 °C. After treatment at 35 and 40 °C the bands corresponding to Cry 4, Cry 10 and Cry11 proteins were observed reduced in A21, X48, R85 isolates.

By another hand, Cyt protein reduction by temperature treatment was observed in A21, X48, R85 and A51 isolates at 35 and 40 °C. In L910 and R84 isolates, the Cyt toxin was degraded only by 40 °C treatment (Fig. 1).

### The effect of chlorine on the larvicidal activity of native isolates

Chlorine had negative effects on the larvicidal activity of *B. thuringiensis* Cuban isolates. Lethal concentrations 50 and 90 were increased significantly in A21, A51, M29, R84, U81 and X48 isolates (Table 2). However, A21 efficiency was improved in chlorinate water. The A51 and M29 were in the same range at both conditions (chlorinated and dechlorinated water). There were not differences in lethal concentrations 50 and 90 of L95 and L910 isolates. The LC_90_ of R89 was reducing significantly in chlorinated water (Table 2).

**Table 2.** Entomopathogenic activity (Lethal Concentration (LC) 50 and 90) and efficiency of isolates of *Bacillus thuringiensis* on *Aedes aegypti* in dechlorinated water and chlorinated water

**Isolates and strains**	**Variable**	**Dechlorinate water 25 °C**	**Chlorinate water 25 °C**
**A21**	LC_50_ (mg/L) (CL)	0.00374 (0.00329–0.00422)	0.02342 (0.02052–0.02707)*
	LC_90_ (mg/L) (CL)	0.01278 (0.0106–0.01609)	0.05545 (0.04470–0.07637)*
	LC_95_ (mg/L) (CL)	0.01810 (0.01455–0.02400)	0.0689 (0.05568–0.10375)*
	Efficiency	4.84	2.79

**A51**	LC_50_ (mg/L) (CL)	0.00153 (0.00135–0.00173)	0.01132 (0.00901–0.01404)*
	LC_90_ (mg/L) (CL)	0.00455 (0.00374–0.00593)	0.04702 (0.03150–0.10350)*
	LC_95_ (mg/L) (CL)	0.00621 (0.00491–0.00855)	0.04700 (0.03730–0.05800)*
	Efficiency	4.05	4.14

**L95**	LC_50_ (mg/L) (CL)	0.09819 (0.08325–0.11700)	0.12627 (0.11321–0.14025)
	LC_90_ (mg/L) (CL)	0.21750 (0.17275–0.30899)	0.23140 (0.19980–0.28970)
	LC_95_ (mg/L) (CL)	0.2724 (0.23088–0.3247)	0.27534 (0.23099–0.3615)
	Efficiency	2.77	2.14

**L910**	LC_50_ (mg/L) (CL)	0.01019 (0.00941–0.01093)	0.01730 (0.01460–0.02122)
	LC_90_ (mg/L) (CL)	0.01920 (0.01757–0.02185)	0.03460 (0.02680–0.05490)
	LC_95_ (mg/L) (CL)	0.02316 (0.0206–0.02706)	0.04157 (0.01849–0.24528)
	Efficiency	2.55	2.42

**M29**	LC_50_ (mg/L) (CL)	0.06569 (0.05833–0.07440)	0.10393 (0.09322–0.11521)*
	LC_90_ (mg/L) (CL)	0.13652 (0.11449–0.17510)	0.21480 (0.18660–0.25910)*
	LC_95_ (mg/L) (CL)	0.1678 (0.1368–0.2250)	0.2639 (0.2237–0.33110)*
	Efficiency	2.56	2.53

**R84**	LC_50_ (mg/L) (CL)	0.00954 (0.00862–0.10358)	0.03412 (0.02797–0.04334)*
	LC_90_ (mg/L) (CL)	0.01913 (0.01741–0.02162)	0.14650 (0.09805–0.28373)*
	LC_95_ (mg/L) (CL)	0.02573 (0.0235–0.0298)	0.16262 (0.14396–0.22360)*
	Efficiency	2.72	5.16

**R85**	LC_50_ (mg/L) (CL)	0.00793 (0.00697–0.00901)	0.01244 (0.01097–0.01425)
	LC_90_ (mg/L) (CL)	0.01700 (0.01478–0.02176)	0.02232 (0.01925–0.03167)*
	LC_95_ (mg/L) (CL)	0.02170 (0.01792–0.02847)	0.02772 (0.02223–0.04034)*
	Efficiency	2.77	2.15

**R87**	LC_50_ (mg/L) (CL)	0.02897 (0.02616–0.03232)	0.03800 (0.03280–0.04600)
	LC_90_ (mg/L) (CL)	0.07831 (0.06713–0.09530)	0.10720 (0.08040–0.16730)
	LC_95_ (mg/L) (CL)	0.10400 (0.0866–0.13102)	0.14250 (0.12460–0.18640)
	Efficiency	3.58	3.75

**R89**	LC_50_ (mg/L) (CL)	0.07146 (0.06444–0.07903)	0.04899 (0.04208–0.05907)
	LC_90_ (mg/L) (CL)	0.17308 (0.14962–0.20820)	0.13740 (0.10310–0.21440)*
	LC_95_ (mg/L) (CL)	0.2223 (0.1873–0.27823)	0.1841 (0.1313–0.31279)
	Efficiency	3.14	3.76

**U81**	LC_50_ (mg/L) (CL)	0.00461 (0.00424–0.00491)	0.01682 (0.01250–0.03050)*
	LC_90_ (mg/L) (CL)	0.00905 (0.00810–0.01043)	0.06720 (0.03501–0.03630)*
	LC_95_ (mg/L) (CL)	0.01095 (0.0096–0.01304)	0.0996 (0.04610–0.74310)*
	Efficiency	2.37	5.9

**X48**	LC_50_ (mg/L) (CL)	0.00213 (0.00192–0.00236)	0.01140 (0.01140–0.01860)*
	LC_90_ (mg/L) (CL)	0.00527 (0.00450–0.00643)	0.05950 (0.03850–0.12500)*
	LC_95_ (mg/L) (CL)	0.00945 (0.00825–0.01127)	0.07182 (0.03585–0.19502)*
	Efficiency	4.44	6.3

**IPS-82**	LC_50_ (mg/L) (CL)	0.00224 (0.00170–0.00273)	0.01803 (0.01440–0.02332)*
	LC_90_ (mg/L) (CL)	0.00567 (0.00457–0.00749)	0.07023 (0.04757–0.13180)*
	LC_95_ (mg/L) (CL)	0.00892 (0.00731–0.01174)	0.12100 (0.08250–0.23779)*
	Efficiency	3.3	6.73

* P≤ 0.05

CL: 95% confidence limits

Efficiency: LC_95_/LC_50_

**Fig. 2.**
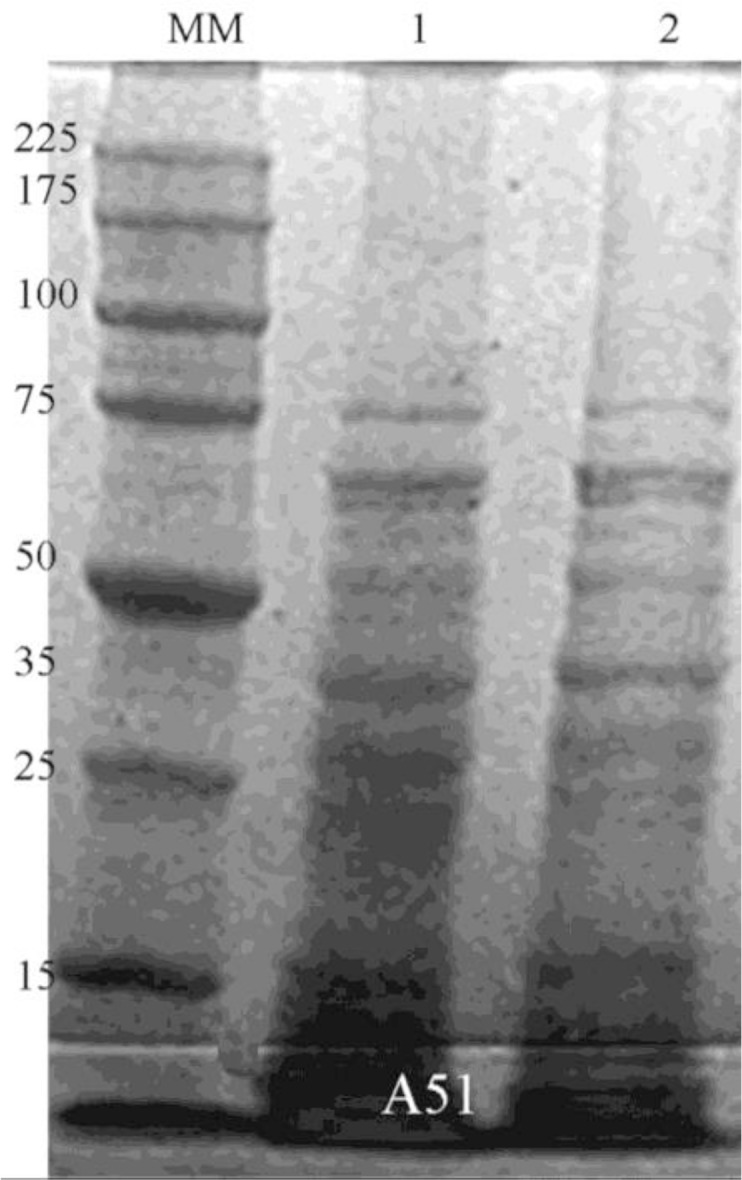
Analysis of Cry and Cyt protein by SDS-PAGE (Coomassie brilliant blue-stained gel) Lane-MM: Molecular weight Marker, and Lanes-1: Proteins profiles of FP without chlorine, Lanes-2: FP exposed to chlorine (2.25mg/L, pH 6.0)

The protein analysis by SDS-PAGE does not reveal visible reduction of the major virulence factors of these isolates (Fig. 2).

## Discussion

*Bacillus thuringiensis* is a viable alternative for insect control due to its specific toxicity against insect larvae. Nevertheless, parasporal crystals activity can be affected by abiotic factors, such as high temperature (18).

In Cuba, the annual temperature average has increased since 1951, and in 1997 and 1998 reached the highest values throughout its history. Overall, the temperature average of the years after 2000 was the warmest of all available climate records (14, 19) increased in dry season, +2.0 °C, and in Jun–Aug: +0.8 °C with higher percentage of days with maximum temperature ≥ 30 °C (20).

Climate variability influences vector population dynamics, distribution and disease transmission (21). Dengue transmission is associated in space and time with local climate effects on survival of its vector *Ae. aegypti*. Thus, more rain and higher temperature generates more transmission (22, 23). Obviously, the biolarvicides should keep high entomopathogen activity at temperatures values over annual average.

Most of our isolates increased their toxicity when temperature raise from 25 to 30 °C and five isolates from 30 to 35 °C. Similar results were obtained with *B. thuringiensis* var. *israelensis* on *Chironomus kiiensis* larvae, results showed changes of LC_50_ doses with a temperatures variation from 15 °C to 30 °C (24). However, no detected differences in larvicidal activity of *B. thuringiensis* (Vectobac AS12, ABG-6164, and AC-513695) against *Culex quinquesfasciatus* with water temperature variations from 15 to 30 °C.

The reduction in LC_90_ in the A21, A51, L910, R84, R85, R89, U81, and X48 isolates, when the temperature increase from 25 to 30 °C may be due to the influence of this factor on the *Ae. aegypti* larvae behavior. The growth of larvae is accelerated with temperature raise, so they begin to feed faster and more toxins Cry and Cyt are ingested (24, 26).

In previous studies, Cry4, Cry10, Cry11 and Cyt proteins were established as the principal virulence factors of Cuban native *B. thuringiensis* isolates (8, 9). Nevertheless we associated the reduction of the Cry and Cyt toxins under then effect of temperature with lack of toxicity of U81 isolate (25–35 °C) and worse efficiency of A21 and A51 isolates. Taking into account that Cyt proteins potentiate the action of Cry (7), the loss of these proteins may be the cause of the significant reduction in the toxicity and efficiency. We also demonstrated reduction of Cry and Cyt proteins at 40 °C but no bioassays were performed. We speculate that in natural breeding sites the toxicity of biolarvicides fail by the loss of these proteins at temperatures over 40 °C. This result should be taken into account for the preservation of *B. thuringiensis* aqueous formulations.

The influence of temperature over Cry proteins has been demonstrated by different authors. The Cry1Ac protein was degraded by exposure to temperature of 35 °C (27). A reduction in Cry protein after 50 °C treatment was demonstrated by SDS-PAGE. They achieved temperature protection of the internal crystals by micro-capsulation (18).

According to our results the isolates, A21, A51, L910, R84, R85 and X48 keep acceptable larvicidal activity at high temperatures, so they would be excellent candidates for the development of formulations better adapted to the climate change effects.

The variability of temperature average, increase the incidence of water and foodborne diseases (20). Chlorine is the most used domestic water disinfectant in the world to prevent these diseases (28), and the mainly breeding sites of *Ae. aegypti* are domestic water containers (29). Therefore, an ideal biolarvicide should keep its activity in chlorinated water.

A biologist from the Cuban Vector Control Programme in Matanzas and Santiago de Cuba provinces referred to the reduction of operational effectiveness of biolarvicides based in *B. thuringiensis* in chlorinated water (personal communications).

In our study, the bioassays showed a significantly increase in CL_50_ in chlorinated water but there are not visible reduction of Cry and Cyt toxins by treatment with chlorine in SDS-PAGE analysis. Chlorine is a non-selective oxidant with a number of effects over the living systems: reacts with a variety of cellular components (30), deactivates enzymatic active sites, decreases the biological functions of proteins and produces deleterious effects on DNA (31). In addition chlorinated waters affect the permeability of the cytoplasmic membrane (32), which could lead to cell death and inhibit the growth of *B. thuringiensis* (33).

Different layers of proteinaceous present in the spore act as a protection from chemical attacks, including oxidizing agents like hydrogen peroxide, sodium hypochlorite, chlorine dioxide, or ozone (34). In case of the chlorine-releasing products produce the lost of refractivity, separation of the spore coats from the cortex, extensive discharge of Ca+, dipicolinic acid, and DNA and finally lysis occurred (35). However, *B. thuringiensis* subs *israelensis* spores are more resistant to chlorine than other spores of either *B. anthracis* or *B. cereus* (33). It may be that *B. thuringiensis* spores resistance to the chlorine effect allows the larvicidal effect in our native isolates when the doses were increases and explain why there were not a significant dose variation in a L_95_, L910 isolates and the increase of the toxicity 90 in R89 isolate.

In spite of the significantly doses variations under chlorine treatment, A21, A51, L910, R85 and X48 isolates had a better larvicidal activity than IPS 82 control strain. For this reason these isolates will be good candidate for *Ae. aegypti* breeding site treatment. Our results have shown the possibility to treat the chlorinated *Ae. aegypti* breeding sites with *B. thuringiensis* based products.

## Conclusion

Our results showed that A21, A51, L910, R85, and X48 isolates have a strong larvicidal activity at 25, 30, 35 °C and chlorinated water. The use of these isolates as biolarvicides can reduce the operational problems in *Ae. aegypti* breeding’s sites.
